# An Ethical Framework for the Use of Horses in Competitive Sport: Theory and Function

**DOI:** 10.3390/ani11061725

**Published:** 2021-06-09

**Authors:** Madeleine L. H. Campbell

**Affiliations:** Department of Pathobiology and Population Sciences, The Royal Veterinary College, Hawkshead Lane, Herts AL9 7TA, UK; mcampbell@rvc.ac.uk

**Keywords:** horse sport, ethics, animal ethics, use of horses in sport, ethical frameworks, social license

## Abstract

**Simple Summary:**

In recent years, there has been increasing societal concern about the use of horses in competitive sport. Ethical frameworks can help stakeholders to make contextual decisions about what should or should not be done in a particular situation. In this paper, it is argued that although there is a recognised need for an ethic (i.e., a set of moral principles relating to conduct) for the use of animals in sport, neither existing animal welfare frameworks nor existing sports ethics frameworks provide us with a suitable or sufficient tool for considering situations in which the athlete is a non-human, non-consenting participant. The theoretical development of a novel ethical framework for the use of horses in sport is presented. The derivation and limitations of the framework are explained. The use of the framework will serve both to underwrite the continuation of the social license to use horses in sport and also to enable those within equestrian sport to critically assess existing and proposed practices and to make welfare-improving adjustments to practice if/where necessary. Practical testing and refinement of the theoretical framework presented in this paper is currently being undertaken in consultation with industry stakeholders, and will be submitted for publication in future.

**Abstract:**

Growing ethical concern about equestrian sport is reflected in publications by regulatory authorities, animal charities, and the lay press; and in government debate and social media. However, attempts by regulators and stakeholders to address ethical issues in equine sport have been discipline specific and ad hoc. Ethical frameworks can help stakeholders to make contextual decisions about what should or should not be done in a particular situation. However, when existing animal welfare frameworks and existing sports ethics frameworks are reviewed in this paper, it becomes clear that none provide us with a suitable or sufficient tool for considering ethical issues which can arise in situations where the athlete is a non-human, non-consenting participant. This paper presents the theoretical development of a novel ethical framework, with the aim of providing stakeholders with a tool which they might apply to the consideration of the ethical questions which inevitably arise in relation to (equestrian) sport. The derivation and limitations of the ethical framework are explained. The use of the framework will serve both to underwrite the continuation of the social license to use horses in sport and also to enable those within equestrian sport to critically assess existing and proposed practices and to make welfare-improving adjustments to practice if/where necessary. The theoretical framework as presented here is currently being practically tested and refined in consultation with industry stakeholders, and that research will be submitted for publication in due course.

## 1. Introduction

Horse sport is important to society in terms of spectator enjoyment, benefits to human mental and physical health, and economic impact. However, sport inevitably exposes horses to (potential) physical and psychological harms. Whilst the use of horses in sport continues to be accepted by the majority of the public, that social license is increasingly tenuous. Public, media, regulatory and governmental unease about health and welfare issues in sporting equines across disciplines, including injury, fatality, ill treatment or neglect, training techniques, transportation, ‘doping’, and their fate after retirement, is growing. Furthermore, concern about the ethical dimension of veterinary sports medicine is increasing amongst veterinarians. 

Whilst the need for the development of an ethic (i.e., a set of moral principles relating to conduct) for the use of animals in sport is anecdotally recognized, to date attempts by regulators and stakeholders to address ethical issues in equine sport have been discipline specific and ad hoc. No coherent interdisciplinary examination has been undertaken to provide an overarching ethical framework which could be applied across equestrian sports to improve practice. 

It is a presupposition of this paper that the use of horses in sport is generally ethically justifiable, but that such use should be constrained by certain specified ‘central tenets’ [1]. The development of an ethical framework for the use of horses in sport represents the application of that approach. I shall start by defining an ethical framework, and go on to consider what the purpose of ethical frameworks is, how they can be structured, and what their limitations are. Having established these broad concepts, I shall go on to consider existing frameworks in animal welfare and human sport. I shall argue that neither existing animal welfare frameworks nor existing human sports ethics frameworks provide us with a suitable or sufficient tool for considering ethical issues surrounding the participation of non-human, non-consenting athletes in sport. A novel ethical framework for the use of horses in competitive sport is therefore proposed, and its limitations explained. This theoretical framework is currently being practically tested and refined in consultation with industry stakeholders, and that research will be submitted for publication in due course.

The aim of developing such a framework is to provide stakeholders—whether they be regulators, organizational committees, owners, trainers, riders/drivers, vets, legislators, members of the public or others—with a tool which they might apply to the consideration of the ethical questions which inevitably arise in relation to (equestrian) sport. It is hoped that the on-going consensual development of this framework will provide stakeholders with a method of addressing ethical issues which can be consistently applied, so promoting transparent and defensible decision and policy making across disciplines (whilst always allowing for inherent differences between sports). Such consistency—providing that it is well founded—can only be in the best interests of the horses involved and indeed of equestrian sport as a whole. 

The purpose of the framework herein proposed is absolutely not to tell any stakeholder what conclusion they ought to be reaching on any particular issue. Rather, the framework is a tool: a logical method which may be used by stakeholders to reach a defensible consensus decision when faced with an ethically challenging scenario. Such use, it is hoped, will serve both to underwrite the continuation of the social license to use horses in sport and also to enable those within equestrian sport to critically assess existing and proposed practices and to make welfare-improving adjustments to practice if/where necessary. 

## 2. A Review of Ethical Frameworks

### 2.1. What Is an Ethical Framework?

An ethical framework is an analytical tool designed to help stakeholders consider the ethical implications of interventions and/or actions [2]. These might include policy proposals, regulations, research initiatives, legislation and direct actions. An ethical framework is not the same as an ethical theory. Whereas ethical theories (for example Deontology, Utilitarianism or Virtue Ethics) are generally self-contained and may conflict with one another, an ethical framework may draw on aspects of several theories and then add in principles of its own to create a systematic approach to addressing an ethical issue. Just as ‘Ethics provides the theoretical basis for assessing why something is good or bad’ [3], so an ethical framework can help us to determine both what should be done and what should not be done in a particular situation. The application of an ethical framework ‘entails identifying actual and potential ethical issues, defining them, determining their scope, (and) specifying criteria for resolving conflicts amongst them’ [4] Thus, ethical frameworks facilitate deliberation, which itself supports and justifies decision making [5]. 

### 2.2. The Purpose of Ethical Frameworks

In all ethical discussions, those participating inevitably start with their own personal and professional moral views. Where these do not align between stakeholders, opinion-based deliberation may become rather circular. This is not helpful when a conclusion needs to be reached in order to implement (or decide not to implement) an action or policy. The advantage of employing an agreed framework to an ethical discussion is that doing so enables us to channel discussion towards a conclusion. A key point about ethical frameworks is that they cannot and do not function to provide stakeholders with one, ‘correct’ answer to an ethical issue. Rather, they provide a rigorous, defensible method for identifying the ethically important aspects of a situation [6]. Frameworks can and should allow for the expression of dissenting opinions, and indeed for the framework itself to be revisited if many of those involved feel that the conclusions which arise from its application are unpalatable. Notwithstanding the inevitable limitation arising from conflicts of interest (see below), if the framework can at least be agreed upon at the outset and logically applied then a foundation exists for building consensus. 

Furthermore, the systematic application of a framework helps those using it not only to apply rigour to their own deliberations but also to explain and defend their decision-making processes. As Kass explains [2], ‘Engaging in the steps of an ethics analysis (should make) us meticulous in our reasoning, requiring us to advocate interventions on the basis of facts and not merely belief. Further, an ethics analysis holds us to high standards, not only for scientific method but also for how respectfully we communicate with and involve constituent communities’. Importantly, the application of an ethical framework allows flexibility for interpretation in different contexts, whilst simultaneously providing justification for decisions made based on the rigour of its application [7].

### 2.3. The Structure of Ethical Frameworks

Most ethical frameworks follow a similar series of steps which might be summarised as follows [4,5]:

- Recognise the ethical issue
- Identify parties (stakeholders) involved
- Gather all relevant information
- Formulate and consider alternatives (using agreed ethical theories or principles)
- Make a decision and reflect upon it
- Act upon the decision and
- Reflect upon the consequences of the action and if necessary review/update the decision.

However, the structures used by different ethical frameworks to work through these steps vary, and may include lists of questions, diagrammatic grids, flow charts, worksheets and labelled diagrams [6]. Thus, as examples, the ethical framework of Kass [2] for public health asks a series of questions having provided some definitions (e.g., of what ‘benefits’ are) in the beginning. Childress et al. [8], also considering public health, developed an ethical framework which provides a series of nine general moral considerations for users to apply. Other frameworks simply provide a set of principles [9]—sometimes boosted by a requirement that they be informed by evidence [10]—or of policy goals to be fulfilled [11]. Checklists are a frequent feature of frameworks [12], whilst adequate deliberation can be promoted by making the framework question based [5].

Whatever the structure of an ethical framework, it should have the capacity to address all aspects of the ethical issue under consideration [6] and should elucidate negative as well as positive considerations [5]. The practical usefulness of an ethical framework is likely to be determined by a combination of applicability and feasibility, and by making it adequately specific rather than too general [4]. ten Have [5] suggests that procedural guidelines for applying the framework can help applicability, whilst criteria should be provided for who should use the framework when. 

### 2.4. Limitations of Ethical Frameworks

Little research has been undertaken to assess the comparative merits of different types of ethical framework, or their effectiveness in practice [6]. Nonetheless, a number of limitations are agreed upon by several authors. Both Hurlimann et al. [4] and ten Have et al. [5] identify lack of specificity as limiting the usefulness of a framework. Whilst the aim of developing a broad framework which may be used by many different people in many different contexts may seem admirable, it is in fact likely to limit uptake of the framework by making it difficult for individuals to see easily how to apply it to their particular situation. Conversely, however, limiting the breadth of an ethical framework can also reduce its utility through narrowing the consideration of those using it. Thus, Manson [6] describes how medical students instructed to apply a ‘Four Principles’ ethical framework were found to ignore considerations not explicitly mentioned in the framework (such as the need to tell the truth) and tended to overlook important discussion of professional and legal guidance. 

A major limitation identified for all ethical frameworks is that they do not provide a mechanism for resolving conflicts between the interests of stakeholders. Although working one’s way through the steps of a framework will help to elucidate what the interests of each stakeholder are, to ensure that none has been forgotten and to describe how possible actions/policies align or not with agreed basic principles, it is frequently nonetheless not possible at the end of the process to identify one ‘correct’ answer to the question being addressed. This is because it is inevitably rare that the interests of all stakeholders will align, meaning that it remains necessary at the end of the deliberation process to make a judgment about which stakeholder’s interests should be given priority. Different people will have different views about that judgement depending on their moral viewpoint and on the ethical theory which they adopt. Thus, the judgments of those using the framework may themselves then conflict. As ten Have et al. state [5] ‘No simple solution seems to be available for dealing with ethical conflicts, although it is precisely the tendency of ethical principles to infringe upon each other that creates the need for frameworks’. Several frameworks incorporate procedures for dealing with differences of opinion. Tannahill [10] encourages an explicit use of a decision-making triangle, and the documentation of judgements. It is suggested that this facilitates discussion about disagreements on the basis of shared principles. Both Kass [2] and Childress et al. [8] argue for some kind of transparent, public process to consider the fairness of proposed outcomes.

Ultimately, individuals may disagree with the conclusion arrived at by working through an ethical framework and such end-stage disagreements may be very difficult to resolve because they arise from fundamental differences of moral view which are informed by experience [6]. They can, perhaps, be mitigated against at the outset of the deliberation process by all participants agreeing upon the theoretical basis (e.g., Utilitarian, Deontological, Virtue Ethics) of the analysis and upon certain central tenets: a strategy which is incorporated into the design of the ethical framework for the use of horses in sport described below. However, the resolution of conflicts is unlikely to be something for which definitive processes can be prescribed within a framework, because both the conflicts and the resolutions rely heavily upon personal and professional experience and judgement [6,8]. This recognition that conflicts will occur and documentation of such occurrence and the reasons for them should be an integral part of using any ethical framework.

## 3. Animal Welfare Frameworks

Whilst there are a number of frameworks designed to assess animal welfare, none of them is an ethical framework in the sense that none address the underlying question of whether the human use of an animal whose welfare is being assessed is morally acceptable. Animal welfare frameworks address ways in which things should be done rather than whether things should be done at all. In this section, a review is provided of the most commonly used of the animal welfare frameworks, along with an explanation of why they fail to function as ethical frameworks.

### 3.1. The 3Rs Framework

The ‘3Rs’ framework was developed as a framework for performing more humane animal research. It centres around the principles of replacement, reduction and refinement [13]. Although the 3Rs framework was developed specifically in relation to the use of animals in science, the principles have application to other uses of animals. For example, in the context of the use of horses in sport the principle of ‘refinement’ is closely related to the idea of avoiding unnecessary harm expounded in [1]: unnecessary harms might be mitigated against by refining factors such as fence design, track surface and training methods. 

The 3Rs framework provides a means of assessing and thereby improving animal welfare, and although it is not in itself an ethical (as opposed to welfare) framework it has become generally accepted in science that failure to follow the 3Rs is unethical because it exposes animals to unnecessarily negative welfare. This is reflected in the way in which the 3Rs are embedded in national and international legislation and regulations on the use of animals in scientific procedures (for example the UK’s Animals (Scientific Procedures) Act 1986 (as amended). However, the 3Rs framework does not itself provide a direct means of answering the question of whether a particular use of animals (in science or elsewhere) is at all ethically permissible. 

### 3.2. The Five Freedoms Framework

This failure to address overriding ethical permissibility is a limitation shared by the Five Freedoms framework which was developed by the Farm Animal Welfare Council (FAWC). The Five Freedoms framework was originally expounded for use in farm animals and originated in the Brambell Report [14]. It was subsequently developed by the British Farm Animal Welfare Council, has been adapted as the basis for the Animal Welfare Act 2006 (England and Wales), the Animal Health and Welfare (Scotland) Act 2006 and the Welfare of Animals (Northern Ireland) Act 2011, and the RSPCA’s advice for horse owners [15]. The concept is referenced in relation to ‘relevant publications’ in Defra’s Code of Practice for the welfare of horses, ponies, donkeys and their hybrids [16].

The Five Freedoms are:

- Freedom from hunger and thirst;
- Freedom from discomfort;
- Freedom from pain, injury and disease;
- Freedom to express normal behaviour;
- Freedom from fear and distress.

It has been suggested by McCulloch [17] that the Five Freedoms are individually necessary and jointly sufficient as a framework for the analysis of animal welfare. However, as McCulloch also notes, the idealism of the Five Freedoms framework makes it an unsatisfactory tool of ethical analysis since it is ‘without power to determine what a satisfactory level of animal welfare is’.

### 3.3. The ‘Five Domains’ Model

This limitation was recognised in the work of Mellor and Reid and later Mellor and other co-workers [18,19,20], who developed a ‘Five Domains model’ of animal welfare which focuses on ‘four physical/functional domains (nutrition, environment, health, behaviour) and one mental domain that reflects the animal’s overall welfare state understood in terms of its affective experiences’ [19]. Incorporated in the Five Domains model [18,19,20] is the important point that the experiencing of temporary negative welfare affects (e.g., thirst) can be important to drive responses which in turn improve welfare through motivating life-sustaining behaviours (in this example, to drink), and that the absolute eradication of all negative welfare effects is not therefore desirable. 

The Five Domains model allows for analysis of negative and positive welfare impacts, and the way in which those interact with each other, to provide an overall assessment of whether the welfare standard being experienced by an animal when taken in is entirety is satisfactory or not. This model provides a comprehensive system of welfare assessment which feeds into quality of life assessment. The Five Domains model as updated [19] is thus more capable than the simple Five Freedoms model of providing comprehensive evidence about the overall level of welfare which an animal is experiencing. 

In relation to horses specifically, a number of welfare assessment frameworks/tools have been developed (reviewed or described, for example, by [21,22,23,24,25,26]). A significant piece of international research aimed at developing equine welfare assessment protocols based on animal based indicators was funded by the European Union as part of its Seventh Framework Programme for Research and published in 2015. The resulting Animal Welfare Indicators (AWIN) welfare assessment protocol for horses [27] focuses on four principles of good housing; good feeding; appropriate behaviour and good health. Much of the subsequent research in this area focused on the refinement of similar indicators, and description of the advantages and limitations of such systems. 

## 4. Animals and Ethical Frameworks

The information provided by tools such as the Five Freedoms framework, the Five Domains model and various welfare assessment tools is information which forms an important evidence base for ethical analysis. However, none of these frameworks, models or tools can themselves provide an answer to the ethical question of what level of welfare is sufficient to allow a particular use of an animal [28], if indeed such a level exists. 

### 4.1. Protocol for Ethical Assessment

The work of Mori et al. [12] attempted to address this deficit in the context of zoo animals, through developing a tool which facilitates ethical assessment of animal welfare concerns. This provides an interesting example in reference to the development of an ethical framework for the use of horses in competitive sport because both are arguably ‘unnecessary’ uses of animals which involve animal harms and benefits to human well-being. The framework of Mori et al. [12] involves gathering evidence about welfare from a number of diverse sources using a variety of techniques, and then superimposing an ethical analysis on those data through the use of a customised ethical matrix. 

### 4.2. Bioethical Matrix

The framework of a ‘Bioethical matrix’ was first proposed by Mepham [29]. Briefly, it involves identifying all relevant stakeholders, and then separating out the stakeholders and chosen ethical theories into a matrix, so that the arguments for and against are contained within cells of the matrix. The ethical theories which Mepham used were autonomy (derived from deontology, i.e., rules-based ethics in which the ‘good’ action is the one in which the established rule is followed), well-being (derived from utilitarianism) and justice (derived from the Four Principles). A typical bioethical matrix construction is illustrated in Table 1.

The bioethical matrix has several weaknesses, most notably that it does not indicate how to weigh the interests of one stakeholder against another; that there are practical limits to the number of stakeholders who can be included; and that (in common with other ethical frameworks) it does not provide a definitive answer. Nonetheless, as a mechanism for ensuring that stakeholders and their interests have been recognised, it performs a useful function. This is explored further in the development of the ethical framework for the use of horses in sport below. 

Mori et al. [12] used their customised ethical matrix to identify ‘The ideal situation of each stakeholder’, and then ‘weighed (that) against that of any other to identify possible internal conflicts’. They then assessed the information contained within the cells of the matrix against a check list of previously agreed policies/principles, e.g., ‘Only a negligible or low risk of welfare health was detected in the risk assessment analysis of physiological parameters’ or ‘The visitor experience analysis detected a positive emotional impact’. Mori et al. [12] argued that use of this framework made up of a combination of data gathering, identification of interests and conflicts and testing against previously agreed principles can help zoos, aquariums, and facilities offering interactive experiences ‘to state and communicate the ethical principles and values that guide them and their commitment to animal welfare…and that)…the use of a uniform protocol (can) help to improve the overall ethical approach and consistency in management decisions.’

## 5. Sport and Ethical Frameworks

Human sports ethics is an established field of applied ethics which is outside the scope of this paper to review, but to which deontological, utilitarian and virtue-based analyses all pertain [30]. The concept of ‘*fair play*’ pervades sports ethics [31,32], and rules relating to drug use and abuse stem from that focus. Integrity is also a commonly occurring theme. In relation to equestrian sport, mention of ‘*fair play*’ and/or integrity feature on the websites, rule books or codes of conduct provided by the publications of Federation Equestre Internationale (FEI), the International Federation of Horseracing Authorities (IFHA), the British Horseracing Authority (BHA), the Hurlingham Polo Association, the Pony Club, the British Equestrian Fededation (BEF), and the International Olympic Committee (IOC). Consistent with such an emphasis on *fair play* and integrity, the principle of justice has been commonly employed in the ethical analysis of sport [31,33,34,35]. The ethical concept of autonomy within sport—particularly of an athlete’s autonomy in decision making about their participation where that may conflict with their objectively perceived welfare [36]—is also an important one.

Within sport, ‘ethical codes’ are used more commonly than ethical frameworks. These differ in that an ethical code is prescriptive, whereas an ethical framework provides a method of analysis. Ethical codes may provide individuals with guidance about how to behave, and may promote awareness of ethical considerations amongst athletes and officials [37], but a code is effectively a rulebook rather than a tool of analysis. The danger of relying upon such codes rather than developing a method of ethical analysis which can be applied to any given situation is that such reliance may inculcate an attitude whereby ‘every action that is not explicitly defined as wrong, will be seen as a viable option’ [38]. 

Examples of ethical frameworks which are broadly applicable to all sports (as opposed to deontological, rules-based ethical codes for individual sports) are comparatively rare. The IOC and United Nations Educational Scientific and Cultural Organisation (UNESCO)—both of which have overarching roles—implicitly rely upon justice frameworks as expounded through their insistence upon ‘*fair play*’ [39,40]. The Australian organisation ‘Play by the Rules’ provides an ethical framework for sport in the form of a check list of reflective questions centred around acquiring facts, applying personal ethics and committing to action [41]. Similarly, teaching materials pertaining to sports ethics provided by the Australian government suggest a method of question-based ethical decision making which focuses on information acquisition, identifying stakeholders, applying personal ethics, identifying weaknesses in one’s own ethical viewpoint, and checking one’s conclusions with somebody else and against the purpose of the sport in which one is participating [42]. 

Knowledge about the theoretical approaches used by those working in the field of sports ethics and of existing codes and frameworks for human sport can be used to inform the development of an ethical framework for the use of horses in sport. However, none of them can appropriately be directly adapted for this use because they do not apply to a situation where the athlete is a non-consenting participant. 

Ethical frameworks are useful tools to help stakeholders determine both what should be done and what should not be done in a particular, contextual situation. For the reasons described above, neither existing animal welfare frameworks nor existing sports ethics frameworks provide us with a suitable or sufficient tool for considering ethical issues surrounding the use of horses in sport. The next part of this paper now goes on to propose such a framework.

## 6. An Ethical Framework for the Use of Horses in Competitive Sport

The aim of developing a novel ethical framework for the use of horses in competitive sport is to provide stakeholders—whether they be regulators, owners, trainers, riders/drivers, vets, legislators, members of the public or others—with a tool which they might apply to the consideration of the ethical questions which inevitably arise in relation to equestrian sport. This framework may be used in international, national or local settings, across equestrian disciplines. The following sections describe the framework, its genesis and limitations. A worked example of how to use the framework is provided in Appendix A.

### 6.1. Structure of the Framework

The framework as here described incorporates structural elements commonly found in ethical frameworks (discussed above (Section 2.3)), and is designed to facilitate (a) ease of access (and therefore uptake) by a variety of stakeholders and (b) transparent decision making. 

**Figure 1:**
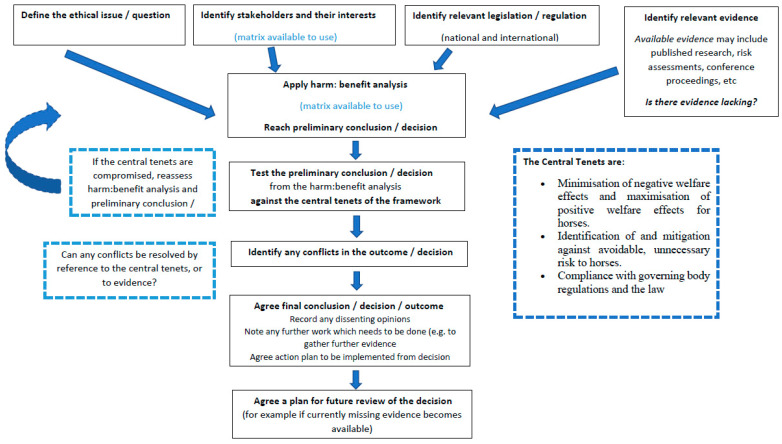
Diagrammatic step by step explanation of how to use the ethical framework.

In line with the work of ten Have et al. [5], which suggested that question-based frameworks promote more adequate deliberation over ethical issues than does providing fixed answers or guidelines, the framework functions in a step-wise fashion to lead users through a question-based analysis. It is presented both in the form of a text-based, detailed discussion of each of the steps (see below) and a flow chart with supporting prompts (Figure 1. This combination of providing a flow chart and a step by step guide to following the process depicted in the flow chart (including suggested use of a ‘stakeholder matrix’) increases accessibility [43] and ease of use by diverse end users [6]. The step by step written description of how to use the framework additionally meets the need to provide ‘procedural guidelines’ described by ten Have et al. [5]. 

The framework relies upon (a) an utilitarian, harm–benefit analysis, and then (b) testing the results of that analysis against the ‘central tenets’ which provide ethical constraints upon the use of animals in sport, described in [1]. These ‘central tenets’ are (a) minimisation of negative welfare effects and maximisation of positive welfare effects, in order to enable horses to have ‘lives worth living’ (b) identification and mitigating against avoidable, unnecessary risk and (c) compliance with governing body regulations and the law. The way in which the framework is structured incorporates consideration of each and all of these elements. The core use of a utilitarian framework facilitates analysis of negative and positive welfare effects (harms and benefits) for each stakeholder. An utilitarian approach is frequently employed to ethical considerations of both animal welfare and human sport, and will be familiar (and therefore easily accessible) to the vast majority if not all of the end users of the framework. Testing the preliminary results of the core utilitarian analysis against the central tenets of (i) identifying and mitigating against avoidable, unnecessary risk and (ii) complying with legislation and regulation introduces both virtue ethics and deontological elements to the analysis. A virtuous person would not intentionally allow exposure to avoidable harms to persist unchanged, whilst an adherence to legislation and regulations is an example of rules-based (deontological) ethics. This combination use of a core utilitarian approach qualified by testing the preliminary results of that analysis against the ‘central tenets’ is consistent with the ‘Core Values’ approach of Manson’s framework [6] which promotes identification of contextually relevant issues in an ethical discussion.

### 6.2. Written Step by Step Description of How to Use the Framework

This section provides a written, step by step description of how to use the framework described in Section 6.1.

**Define the Ethical Issue / Question**

- What is the ethical question/the issue which requires an answer / decision?
- What is the scope of the question?

**Identify Stakeholders and Their Interests**

Examples of stakeholders who might be relevant include (NB this list is not exhaustive, and not all stakeholders given as examples here may be relevant to every use of the framework in practice).

Equine stakeholders

- Those directly (actively) involved in the sport
- Those indirectly involved in the sport, e.g., young horses not yet in training; retired horses; broodstock; future generations who might be affected by the decision (e.g., through genetic effects)
- Horses not involved in the sport (if there are relevant ‘knock on’ consequences)

Human stakeholders

- Horse owners
- Horse breeders
- Horse riders/jockeys/drivers (‘athletes’)
- Those directly employed by or with a business interest in the sport
- Those indirectly employed by or with an indirect business interest in the sport
- Veterinary surgeons and other members of the ‘veterinary team’
- Members of the public with an active interest in the sport (e.g., spectators/those engaged in betting)
- Members of the public with no particular interest in the sport but a general interest in animal welfare
- Regulators
- Policy makers
- Law makers
- Animal charities
- Lobbying organisations
- Media

Other stakeholders

- The environment

**Assess the relevant evidence**

What evidence about the issue under consideration is available?

- Peer-reviewed journal papers
- Non-peer-reviewed papers, books, and reports
- Peer-reviewed or non-peer-reviewed papers which are not about the issue under consideration but are about a related issues (for example in other species, or other sports)
- Expert opinion
- Stakeholder opinions (e.g., from publications; conference proceedings, websites)

Consideration should be given to the quality of evidence [44]. Whilst a ‘hierachy of evidence’ is recognised [44], the relative weighting of different types of evidence is the responsibility of those using the framework, and will be context dependent.

- What evidence about the issue under consideration is lacking/how could this be obtained?

**Identify relevant legislation/regulation**

- International legislation
- National legislation
- Sport specific regulations (which may include international or national variation)

**With reference to the interests of each stakeholder and considering also the severity of impact on stakeholders (for example in terms of intensity, duration and basic needs) apply a harm–benefit analysis to the question/issue**

Use of a ‘stakeholder matrix’ such as this one (Table 2) may help to focus this consideration:

**Reach preliminary conclusion/decision based on the harm–benefit analysis**

**Test preliminary conclusion/decision against the central tenets**

The central tenets of the framework are:

- Minimisation of negative welfare effects and maximisation of positive welfare effects for horses.
- Identification of and mitigation against avoidable, unnecessary risk to horses.
- Compliance with governing body regulations and the law

If any of the central tenets are compromised by the preliminary conclusion/decision reached through the harm–benefit analysis, reassess both the analysis and the conclusion.

Note: There may be occasions on which the initial conclusion is not compliant with current regulation/legislation and when—having reassessed both the analysis and the conclusion—the users of the framework still believe that their conclusion is correct and that current regulation/legislation needs reviewing. If this occurs it should be explicitly stated and recorded.

Note: this testing against central tenets will assist in ‘weighing’ different stakeholder interests if a particular conclusion/decision would, in a harm–benefit analysis, be to the overall benefit of one stakeholder and the detriment of another. For example, a particular preliminary conclusion/decision from the harm–benefit analysis might provide a substantial economic benefit to many humans but involve the acceptance of an identifiable risk to equine welfare which could be mitigated against. In that case, implementing the preliminary conclusion/decision would contravene one of the central tenets of the framework (‘Identification of and mitigation against avoidable, unnecessary risk to horses’). This would indicate that the weighting of interests in the conclusion of the harm–benefit should be shifted in favour of equine (not human) interests, and the conclusion adjusted accordingly. Thus, testing initial conclusions from the harm–benefit analysis against the central tenets is a balancing and rebalancing process. The framework deliberately does not say anything about the relative weighing of different (sometimes conflicting) interests amongst humans—that must be left to the users of the framework, with appropriate acknowledgment of conflicts where they occur (see below).

**Identify any conflicts in the conclusion/decision.**

Conflicts may occur between stakeholder interests, or in the acceptance amongst those using the matrix of the conclusion/decision which has emerged from its employment.

- Can any conflicts be resolved by further reference to the central tenets of the framework (see ‘testing against central tenets’ above)?
- Can any conflicts be resolved by reference to evidence? Sometimes, apparent conflicts of interest are in fact disagreements over facts, and can be resolved by elucidation of those facts or by gathering further evidence (for example, about the extent or nature of a harm).
- It is to be expected that conflicts will occur [4,8]. Where this happens and they cannot be resolved they should be noted, along with a brief explanation of the reason why they cannot be resolved (e.g., insufficient evidence to reach a definitive conclusion; disagreement about weighing interests).

**Agree final conclusion/decision/outcome**

- Record any dissenting opinions
- Note any further work which needs to be done (e.g., to gather further evidence)
- Agree an action plan to be implemented as a consequence of the conclusion/decision

**Agree a plan for future review of the decision**

For example, if a lack of evidence has been identified as a factor limiting the validity of the decision, make a plan for commissioning appropriate research/tracking the publication of relevant evidence and reviewing the decision when the evidence does become available.

### 6.3. Visual Representation of How to Use the Framework

This section provides a visual guide to how to use the framework, in the form of a flow chart (Figure 1). 

A worked example of applying the framework for the use of horses in competitive sport to a sample question is provided in Appendix A.

## 7. Conclusions

The ethical framework for the use of horses in sport presented above consists of a core utilitarian analysis which is qualified by virtue ethics and deontological elements, and by testing of preliminary conclusions against specified ‘central tenets’. The framework provides stakeholders in equestrian sport with a tool which they may use in a step-wise fashion to analyse any ethical issue which arises. The ethical framework for the use of horses in sport is deliberately designed to be applicable to all equestrian sports. However, as Hurlimann et al. highlight [4], ethical frameworks which are over-general are unlikely to be helpful in practice as their very generalisability makes it harder for stakeholders to easily see the applicability to their own situation. It is therefore anticipated that individual equestrian sports may choose to use this ethical framework as a starting point, and subsequently to develop sport-specific codes of ethics. This is likely to be a reiterative process, whereby application of the framework to particular issues may elucidate a generalizable rule which stakeholders feel could be usefully written into a code of ethics, and a code of ethics (once it exists) feeds back into the application of the framework through providing regulatory evidence which is used in deliberations. This process and any limitations which the generalizable nature of the framework cause could be the subject matter of future research. 

## Figures and Tables

**Table 1:** Example of a typical bioethical matrix construction.

Stakeholder	Autonomy	Well-being	Justice
e.g., Animal			
e.g., owner			
e.g., Vet			
e.g., public (consumer)			
e.g., public (opinion)			

**Table 2:** A sample ‘stakeholder matrix’. The use of such a stakeholder matrix is demonstrated in the worked example provided in Appendix A.

Stakeholder	Harms Associated with the Action/Decision	Benefits Associated with the Action/Decision
1		
2		
3		
4		
etc.		

## Data Availability

Not applicable.

## Appendix A. A Worked Example of Using the Ethical Framework for the Use of Horses in Sport

Recently, there has been interest in the lay equine press in the question of whether all horses being kept in a stable require daily turnout. Whilst such equine management is legislated for in some countries, in others. Daily turnout is a recommendation rather than a requirement. This appendix uses that issue as an example question with which to demonstrate the use of the framework. The purpose of providing this worked example is not to suggest a ‘correct’ answer to this particular question, but simply to demonstrate how the framework itself could be used to approach *any* ethical question. Instructions are written in black, with possible answers in blue. These answers are illustrative, and by no means exhaustive.

**Define the ethical issue/question**

- What is the ethical issue/question which requires a policy formulation/decision?

The more general question of whether all horses kept in stables should be turned out for free exercise is narrowed to apply to competition horses only, since this is an ethical framework for the use of horses in sport. Thus, the ethical questions is determined to be: ‘Should it be a legal requirement that all horses primarily used for competitive purposes which are kept in stables be turned out for free exercise on a daily basis?’

NB this does not apply to horses which are being kept stabled under veterinary advice (e.g., due to injury).

NB ‘competition’ is defined as any horses involved in any equestrian sport, or being raised for a future in or retired from such involvement.

NB ‘turnout’ in this context is being considered purely in relation to the ability to exercise and express normal behaviours. For ease of analysis, it is assumed that adequate nutrition will be provided whether or not the horse is turned out.

- What is the scope of the question?

Could also consider whether horses should always be turned out with company (either equine other animal) or whether it is acceptable to turn them out alone.

Could also consider whether horses of differing ages and sexes have different needs.

(For reasons of space, these supplementary questions will not be considered in detail in the worked example).

**Identify stakeholders and their interests**

Examples of stakeholders who might be relevant include

- Equine stakeholders

All competition horses at all stages of life who are being kept stabled.

- Human stakeholders
  o Horse owners
  o Horse breeders
  o Those directly employed by or with a business interest in equestrian sport for which horses are kept for competition, e.g., grooms, riders, trainers, regulators for a given sport.
  o Those indirectly employed by or with an indirect business interest in equestrian sport for which horses are kept stabled or kept outside of stables, e.g., manufacturers of stables; bedding suppliers; fencing suppliers; muck heap removal services.
  o Veterinary surgeons and other members of the ‘veterinary team’
  o Members of the public with an active interest in the sport (e.g., spectators/those engaged in betting)
  o Members of the public with no particular interest in the sport but a general interest in animal welfare
  o Regulators
  o Policy makers
  o Law makers
  o Animal charities
  o Lobbying organisations
  o Media
- Other stakeholders
  o ‘The environment’ (impact via poaching of fields, muck removal, water supply, etc.).

**Assess relevant evidence**

- What evidence about the issue under consideration is available?
  o Peer-reviewed journal papers

There is extensive literature about the relationship between being stabled and the development of abnormal (stereotypic) behaviours in horses, e.g., [45,46,47,48,49,50,51]. However, the development of stereotypies may be associated with motivation to chew and forage rather than turn out *per se* [52,53].

Providing exercise, including in the form of turnout, generally reduces behaviours which are undesirable during standard handling/training situations [54].

The relationship between keeping horses either stabled or turned out and the incidence of injury and disease [50] is complex since it involves not only physiological but also psychological and social processes. For example, lack of access to grazing is generally thought to increase the prevalence of the development of squamous stomach ulcers, but region and management are also important factors and for some individual horses stress associated with pasture mates may offset the benefit of having access to pasture [55].

There is published literature to support the idea that human-controlled or at liberty exercise in young horses offers a protective effect against some forms of orthopaedic disease and fatality due to injury [56,57,58,59,60,61,62]. The protective effect of exercise during turnout is likely.

o Non-peer-reviewed papers, books, and reports

A number of such articles exist which in recent years have tended towards recommending daily turnout whilst recognising the risks of injury which can be associated with it, e.g., [62,63,64].

o Peer-reviewed or non-peer-reviewed papers which are not about the issue under consideration but are about a related issue (for example in other species, or other sports)

See notes above re: peer-reviewed articles on the protective effect of controlled exercise.

o Expert opinion

The Code of Practice for the Welfare of Horses, Donkeys and Hybrids [16] recommends that horses should be turned out.

o Stakeholder opinions (e.g., from publications; conference proceedings, websites)

Several websites of equine welfare charities/organisations recommend daily turnout whilst recognising the risks of injury which can be associated with it, e.g., https://www.worldhorsewelfare.org/advice/management/stabling (accessed on 7 June 2021)

https://www.rspca.org.uk/adviceandwelfare/pets/horses/behaviour (accessed on 7 June 2021)

https://www.bhs.org.uk/advice-and-information/horse-care/winter-care (accessed 7 June 2021)

- What evidence about the issue under consideration is lacking/how could this be obtained?

Information about incidence and causes of disease and injury in horses which are kept stabled except when undertaking controlled exercise compared to those which have a daily opportunity to exercise freely is lacking. Research is necessary to provide this information.

**Identify relevant legislation/regulation**

- International legislation
- National legislation
- Sport specific regulations (which may include international or national variation)

International legislation:

*OIE* standards on *animal welfare do not address competition horses (though they do address ‘working equids’).*

Animals used in competitions are excluded from EU Treaties.

In Switzerland, the Animal Welfare Ordinance of 2008 https://www.globalanimallaw.org/downloads/database/national/switzerland/TSchV-2008-EN-455.1-2011.pdf (accessed 7 June 2021) dictates that working horses (those ridden or worked regularly) must be allowed free time in open outdoor areas at least two days a week for at least two hours each time. Unworked horses (e.g., retired horses or broodmares) must have at least two hours of outdoor free time every day. Young horses (up to two-and-a-half years old) must be kept in groups.

Danish legislation (2008) dictates that all horses should have a minimum of 2 h in a paddock or ‘other exercise’ every day. Paddocks should have free choice shelter from weather, sun, insects, with compatible companions.

National legislation (for the purposes on this worked example we concentrate on UK legislation):

The Animal Welfare Act (2006) (England and Wales), the Animal Health and Welfare (Scotland) Act 2006 and the Welfare of Animals (Northern Ireland) Act 2011 require owners and keepers to ensure that horses’ needs to express normal behaviours are met.

The Code of Practice for the Welfare of Horses, Donkeys and Hybrids [16] suggests that ‘All stabled horses, apart from those on box rest for veterinary reasons, will benefit from daily turnout’

Sport specific regulations (which may include international or national variation)

None found

With reference to the interests of each stakeholder and considering also the severity of impact on stakeholders (for example in terms of intensity, duration and basic needs) apply a harm–benefit analysis to the question/issue

Horses which are kept stabled all of the time except when being exercised under the control of a human are likely to suffer chronic, low-moderate level harms (e.g., boredom; stiffness, chronic respiratory disease) and may sometimes suffer more acute, higher intensity harms (e.g., colic). Horses which are turned out daily (specially if weather conditions are inclement) may suffer chronic, low level harms (e.g., ‘mud fever’) or acute, more intense harms, e.g., orthopaedic injury. See matrix below.

Basic needs of horses include

- Freedom from discomfort
- Freedom from pain, injury and disease
- Freedom to express normal behaviour

Use of a ‘stakeholder matrix’ such as this one may help to focus this consideration:

NB in this example, for reasons of space, the matrix has not been comprehensively completed—example key stakeholders only have been included, for the purposes of illustration.

**Stakeholder**	**Harms Associated with the Action/Decision**	**Benefits Associated with the Action/Decision**
1. Horses	Being kept stabled restricts the freedom to express normal behaviours and to move normally, and may result in stereotypic behaviours/‘stable vices’. Being kept stabled is associated with an increased incidence of some diseases, e.g., respiratory disease; colic; thrush. Daily turn out may cause injuries, e.g., through entanglement in fencing, uncontrolled exercise or interaction with other horses. Weather conditions, e.g., excessive heat or rain may result in discomfort during turnout. Insects can irritate horses and can be associated with disease, e.g., insect born diseases or allergic skin diseases. Turnout is often associated with access to pasture and this may be contraindicated in some horses, e.g., those prone to laminitis. Individual horses who are not used to being turned out may find it stressful–acclimatisation is necessary.	Daily turnout facilitates expression of normal behaviours and freedom of movement. Daily turnout *may* help to reduce the risk of orthopaedic injury during training and competition by keeping animals supple and building muscle strength and co-ordination
2. Owners	Variable financial impact of keeping horses stabled versus stabled plus daily turnout. Being kept stabled may result in increased veterinary costs for issues such as respiratory disease. Daily turnout could increase veterinary costs IF more injuries resulted. IF more injuries resulted from turnout then the competitive career and value of horse could be negatively affected. In some parts of the world it is difficult to provide suitable turn out during very wet winters. If weather conditions are inclement horse may be more prone to conditions such as ‘mud fever’, resulting in increased veterinary costs Owners may feel that regulation requiring daily turnout of horses is ‘interference’ in their autonomous right to look after their property as they see fit.	Horses may be less stressed as a result of daily turnout, with positive effects, e.g., reduction in gastric ulceration (which reduces veterinary costs and improves competitive performance). IF daily turnout is associated with a protective effect against orthopaedic injury/disease then the competitive career and value of horse could be positively affected. Owners may ‘feel good’ about their horses having the freedom to exhibit more natural behaviours.
3. Grooms	Horses which are turned out daily are likely to need more grooming; increased work associated with wet rugs and leading horses to and from fields, supplying feed in field, etc.	Less time stabled may reduce workload of mucking out. Grooms may ‘feel good’ about their horses having the freedom to exhibit more natural behaviours Horses may be easier to handle if they have time to exercise freely
4. Rider	Having horses turned out may be less convenient and more time consuming than having them readily accessible in a stable.	Horses who have been relaxing and moving around during turnout may be more easily trainable and require less warm up time than those who are always kept stabled.
5. Public with an interest in animal welfare		Interested in animals’ freedoms to express normal behaviours being protected. May feel that voluntary adoption of good practice is insufficient and that legislation is necessary to safeguard animal welfare.
6. Policy makers (government)	Government may have an ideological objection to ‘interfering’ in animal owners’ autonomous decision making processes. Legislation may not necessarily be the most effective means of effecting desirable changes in equine management processes. Legislation is only effective if enforced, which requires financial commitment.	Animal welfare is generally a vote winning (or losing) issue in some countries. Governments may thus have a pragmatic interest in visibly driving national animal welfare laws which reflect public attitudes towards animal welfare. Governments may have an ideological commitment to safeguarding animal welfare.
Etc.		

**Summary of harm–benefit analysis**

Daily turnout for stabled competition horses is associated with clear benefits to psychological animal welfare and behaviour. Daily turnout may also be associated with some risks to animal welfare through turn-out associated injury and disease. Unlimited access to pasture can also result in disease in some individuals. However, being kept stabled is also associated with (different) risks to health and therefore welfare, and additionally has a negative psychological effect on welfare.

Allowing for uncertainty arising from the weighing of these risks and benefits to equine health associated with being kept stabled versus being turned out, the advantages to all human stakeholders of keeping horses stabled are primarily those of convenience, and the disadvantages of daily turn out are primarily those of inconvenience (which may be associated with increased staff costs).

There are precedents showing that any concerns on the part of animal owners about contravention of their autonomy and right to make decisions about how they keep their property (animals) may be overridden by animal welfare interests—animal welfare legislation already very clearly establishes limitations to ways in which owners may treat animals, and these limitations are supported by welfare codes of practice.

‘The public’ has an interest in animal welfare being safeguarded and would therefore support systems which enable the expression of normal behaviours.

Policy makers have an interest in regulations pertaining to horses being consistent with national and international animal welfare laws and reflecting public attitudes towards animal welfare and are therefore likely to support systems which enable the expression of normal behaviours.

**Reach preliminary conclusion/decision based on the harm–benefit analysis**

There is a lack of evidence about rates of injury and disease in horses kept stabled versus those kept stabled with daily turnout, which needs to be addressed.

Daily turnout may be associated, through impact on injury and disease and stress, with some negative welfare effects for individual horses.

There is clear evidence that keeping horses stabled all of the time has a detrimental effect on welfare by limiting opportunities for normal behaviours, including foraging and social interactions. Daily turnout is likely to be associated with positive psychological welfare effects for the vast majority of horses.

Based on current evidence, it therefore seems likely that daily turnout of competition horses would improve equine welfare overall and be consistent with the interests of members of the public who are interested in animal welfare, and of government. Harms to owners/riders/grooms do exist, but are largely those of inconvenience and therefore outweighed by the benefits to horses, the public and governments.

Improvements in animal welfare may be effected either through legislation or regulation or voluntary adoption of recommendations. It is suggested that laws which positively state what a person must do are most effective in terms of improving animal welfare [65]. However, any legislation is only effective if enforced.

Preliminary conclusion: The majority of stabled competition horses’ welfare is enhanced by being turned out daily. Legislation requiring such management would therefore be appropriate. However, (a) such legislation must be sufficiently flexible to allow for derogations for particular animals, e.g., if a health condition makes turnout inappropriate or if the horse’s prior management and social conditioning is such that turn out is very stressful for the animal concerned and (b) the effectiveness of adopting such a policy is dependent upon having the resources to enforce any new legislation.

**Test against the central tenets**

The central tenets of the framework are:

- Minimisation of negative welfare effects and maximisation of positive welfare effects for horses.

As described above, daily turnout may be associated, through impact on injury, disease and stress with some negative welfare effects for individual horses. However, daily turnout is likely to be associated with positive physical and psychological welfare effects for the vast majority of horses. The preliminary decision is therefore consistent with this tenet.

- Identification of and mitigation against avoidable, unnecessary risk to horses.

Many of the risks associated with daily turnout, e.g., of injury due to fencing and interaction with other horses and disease due to muddy conditions can be mitigated against by management systems. Such mitigation should recognise, for example, the fact that safety may be increased by turning some horses out by themselves (e.g., stallions, or if one horse persistently kicks other horses); and that horses who are not used to being turned out may find it initially stressful and should be introduced to turn out for short, increasing periods.

The preliminary decision is therefore consistent with this tenet, and considered application of this tenet in fact shifts the harm–benefit analysis further in the direction of concluding that daily turnout should be a legal requirement.

- Compliance with governing body regulations and the law

The preliminary decision is consistent with existing legislation, e.g., the

The Animal Welfare Act (2006) (England and Wales), the Animal Health and Welfare (Scotland) Act 2006 and the Welfare of Animals (Northern Ireland) Act 2011. Specific regulation/secondary legislation may be necessary to implement the conclusion of this analysis.

- If any of the central tenets are compromised by the preliminary conclusion/decision reached through the harm–benefit analysis, reassess both the analysis and the conclusion.

Not necessary

**Identify any conflicts in the conclusion/decision.**

Conflicts may occur between stakeholder interests, or in the acceptance amongst those using the matrix of the conclusion/decision which has emerged from its employment.

- Can any conflicts be resolved by further reference to the central tenets of the framework? (see ‘testing against central tenets above)

Conflicts between the needs of horses and the convenience-bases interests of owners/grooms/riders may be resolved through application of the tenets, as described above.

There is a conflict between owners’ autonomous rights to make their own decisions about how their property is managed, and the requirement to meet animals’ basic needs. Childress et al. [8] suggest that where conflicts of interest exist the right to autonomy has to be qualified—giving rise to a principle of ‘least infringement’.

Conflicts inherent within the interests of horses, e.g., risk of disease higher if kept in, risk of injury higher if turned out, may be reduced through application of the tenets, as described above.

- Can conflicts be resolved by reference to evidence? Sometimes, apparent conflicts of interest are in fact disagreements over facts, and can be resolved by elucidation of those facts or by gathering further evidence (for example, about the extent or nature of a harm)

Potential conflicts, e.g., increased veterinary costs for owners associated with turn out versus improved psychological welfare for horses associated with turnout may be resolved in future by acquisition of further evidence about incidence and causes of disease and injury in horses which are kept stabled except when undertaking controlled exercise compared to those which have a daily opportunity to exercise freely.

**Agree final conclusion/decision/outcome**

- Record any dissenting opinions
- Note any further work which needs to be done (e.g., to gather further evidence)

Further work needed to gather evidence about the risks to equine health associated with being turned out and stabled. Note that these risks may differ for individual horses, depending on temperament, previous experience and health status.

Further work needed to provide evidence about the optimal minimum time of daily turnout for horses—it is not clear where such specifications as exist in international legislation originate.

- Agree an action plan to be implemented as a consequence of the conclusion/decision

Decision that daily turnout for competition horses should become a legal requirement agreed. However, such legislation must be sufficiently flexible to allow for derogations to meet the health and social needs of particular animals. Further work is now needed to elucidate the most effective means of effecting desirable changes in equine management processes (e.g., secondary legislation) and to determine what minimum daily time period of turn out (if any) should be specified.

**Agree a plan for future review of the decision**

For example, if a lack of evidence has been identified as a factor limiting the validity of the decision, make a plan for commissioning appropriate research/tracking the publication of relevant evidence and reviewing the decision when the evidence does become available.

Publication of evidence about the risks to equine health associated with being turned out and stabled to be tracked. Decision to be reviewed in light of future publications.

Before the policy recommendation can be implemented, further work is needed to elucidate the most effective means of enacting this policy recommendation (e.g., through secondary legislation).

Before the policy recommendation can be implemented, the basis of minimum daily time period of turn out (if any) which would be specified in legislation needs to be explained.
